# Supplementary material for: Decision-making in acute viral bronchiolitis: A universal guideline and a publication gap
Source: PLoS One. 2020 Aug 18;15(8):e0237801. doi: 10.1371/journal.pone.0237801 (PMC7433885; doi:10.1371/journal.pone.0237801)
Supplement: S1 Table — Close of database was October 1st 2019. (DOCX) [file pone.0237801.s001.docx]

S1 Table. List of published (n=50) and unpublished (n=19) trials in acute viral bronchiolitis. Close of database was October 1st 2019.

| Trial number | published | Date of completion [DD.MM.YY] | Date of publication  [DD.MM.YY] | Time to Publication [months] | Study Title | Interventions | Patients enrolled [n] | Country |
| --- | --- | --- | --- | --- | --- | --- | --- | --- |
| NCT00076973 | yes | 01.10.06 | 26.06.08 | 20 | An Investigational Drug Study to Treat Respiratory Symptoms Associated With Respiratory Syncytial Virus (RSV) Bronchiolitis (0476-272) | Drug: montelukast sodium\|Drug: Comparator: placebo | 1125 | U.S. |
| NCT00116584 | yes | 01.10.11 | 01.12.11 | 2 | Heliox-Driven Racemic Epinephrine Nebulization in Treatment of Moderate to Severe Bronchiolitis in Pediatric ED Patients | Drug: heliox | 72 | U.S. |
| NCT00119002 | yes | 01.04.06 | 26.07.07 | 15 | The Effectiveness of Oral Dexamethasone for Acute Bronchiolitis | Drug: dexamethasone\|Drug: Placebo | 598 | U.S. |
| NCT00122785 | yes | 01.09.04 | 01.07.07 | 34 | Single Injection of Dexamethasone for Acute Bronchiolitis in Young Children | Drug: dexamethasone | 170 | Thailand |
| NCT00125450 | yes | 01.02.08 | 28.09.10 | 31 | Evaluation of Chest Physiotherapy for Acute Bronchiolitis in Toddlers (BRONKINOU) | Procedure: Chest Physiotherapy with Forced Expiratory Technique\|Procedure: Nasopharyngeal Aspiration | 500 | France |
| NCT00151905 | yes | 01.09.06 | 01.09.07 | 12 | The Use of an Inhaled Salt Solution to Treat Viral Lung Infections in Infants. | Drug: 3 % hypertonic saline | 96 | Canada |
| NCT00213226 | yes | 01.04.06 | 01.09.08 | 29 | Single Dose Versus Multiple Doses of Dexamethasone in Children With Acute Bronchiolitis | Drug: dexamethasone | 120 | Canada |
| NCT00261937 | yes | 01.04.06 | 01.03.08 | 23 | Inhaled Furosemide Versus Placebo for Acute Viral Bronchiolitis in Hospitalized Infants | Drug: Inhaled Furosemide vs Placebo (Normal Saline) | 32 | Israel |
| NCT00394069 | yes | 01.12.03 | 01.06.06 | 30 | PK Study in 3- to 6- Month-Old Children (0476-268)(COMPLETED) | Drug: MK-0476, montelukast sodium | 14 | U.S. |
| NCT00394160 | yes | 01.10.05 | 01.04.08 | 30 | Safety and Pk Study in 1- to 3- Month-Old Children With Bronchiolitis (0476-297) | Drug: MK0476, Montelukast Sodium /Duration of Treatment : 14 Days | 12 | U.S. |
| NCT00513890 | yes | 01.02.10 | 01.01.13 | 35 | Interest of Non Invasive Ventilation Versus Oxygen During the Intial Managment of Infant With Bronchiolitis | Device: Infantflow [EME, Brighton, England] | 19 | France |
| NCT00524693 | yes | 04.11.08 | 01.12.08 | N/A | Montelukast in Acute RSV Bronchiolitis | Drug: Montelukast\|Drug: Placebo granules | 51 | Israel |
| NCT00619918 | yes | 01.05.11 | 01.10.14 | 41 | Nebulized Hypertonic Saline for Bronchiolitis | Drug: Nebulized 3% saline\|Drug: Placebo | 447 | U.S. |
| NCT00622817 | yes | 01.03.05 | 01.01.10 | 58 | The Influence of Inhaled Adrenalin Versus Decongestant as a Local Nasal Treatment in Bronchiolitis | Drug: xylometazoline HCL 0.05%\|Drug: Epinephrine 1mg | 65 | Israel |
| NCT00677066 | yes | 01.12.07 | 16.10.08 | 10 | Safety Study of Home Oxygen Therapy for Children With Acute Bronchiolitis | Procedure: Home oxygen therapy | 44 | Australia |
| NCT00677729 | yes | 01.05.09 | 01.11.10 | 18 | Hypertonic Saline to Reduce Hospital Admissions in Bronchiolitis | Drug: solution contains 1 mg salbutamol plus 3% hypertonic saline\|Drug: solution contains 1 mg salbutamol plus 0.9% saline | 81 | Canada |
| NCT00884429 | yes | 01.04.10 | 01.06.12 | 26 | Effectiveness of Chest Physiotherapy in Infants With Acute Viral Bronchiolitis | Procedure: Chest physiotherapy - Conventional\|Procedure: Chest physiotherapy - actual techniques\|Procedure: 3-Airway suction | 33 | Brazil |
| NCT01016249 | yes | 01.04.09 | 01.06.10 | 14 | Nebulized 5% Hypertonic Saline for the Treatment of Bronchiolitis | Drug: Treatment 1. (5% Hypertonic saline + Epinephrine)\|Drug: Treatment 3. (3% Hypertonic saline + Epinephrine)\|Drug: Treatment 2. (Normal saline + Epinephrine) | 187 | Qatar |
| NCT01065272 | yes | 01.08.12 | 16.09.13 | 13 | Dexamethasone in the Treatment of Bronchiolitis in Patients With Either Eczema and or Family History of Asthma | Drug: Dexamethasone\|Drug: Placebo | 200 | Qatar |
| NCT01120496 | yes | 01.11.09 | 01.12.11 | 25 | Nebulized Hypertonic Saline Treatment in Hospitalized Children With Moderate to Severe Viral Bronchiolitis | Drug: 3% hypertonic saline\|Drug: normal saline | 135 | China |
| NCT01189149 | yes | 01.03.12 | 01.03.13 | 12 | Intravenous Fluids Versus Naso/Orogastric-tube Feeding in Hospitalized Infants With Bronchiolitis | Procedure: IV fluids\|Procedure: Naso/oro gastric tube feedings | 35 | Israel |
| NCT01238445 | yes | 01.11.16 | 26.08.12 | N/A | Assessing Response to Albuterol in Bronchiolitis |  | 29 | U.S. |
| NCT01247064 | yes | 01.12.11 | 01.07.14 | 31 | Hypertonic Saline for Acute Bronchiolitis | Drug: Nebulized 3% saline\|Drug: Nebulized 0.9% Normal Saline | 62 | U.S. |
| NCT01276821 | yes | 01.04.11 | 10.09.15 | 53 | Efficacy of Nebulised Hypertonic Saline (3%) Among Children With Bronchiolitis. | Drug: L-Epinephrine and Normal Saline (0.9%)\|Drug: L-Epinephrine and Hypertonic Saline (3%) | 100 | Nepal |
| NCT01300325 | yes | 01.12.10 | 01.04.12 | 16 | Effectiveness of Nebulized Hypertonic Saline and Epinephrine in Hospitalized Infants With Bronchiolitis | Other: 3% hypertonic saline solution | 136 | Italy |
| NCT01383655 | yes | 01.01.16 | 07.02.18 | 25 | Intravenous Magnesium in Wheezy Bronchitis | Drug: Magnesium Sulfate\|Drug: Placebo | 61 | Finland |
| NCT01435486 | yes | 01.11.14 | 01.10.16 | 23 | Caffeine Citrate for the Treatment of Apnea Associated With Bronchiolitis in Young Infants | Drug: Caffeine citrate\|Drug: Normal saline | 90 | Qatar |
| NCT01441466 | yes | 01.05.12 | 01.12.13 | 19 | Cohort Isolation and Cross-infection in Bronchiolitis | Other: Isolation | 48 | Netherlands |
| NCT01469845 | yes | 01.01.14 | 01.12.14 | 11 | The SABRE Trial of Hypertonic Saline in Acute Bronchiolitis | Device: 3% hypertonic saline | 300 | U.K. |
| NCT01488448 | yes | 01.08.15 | 01.12.15 | 4 | A Study of Hypertonic Saline for Infants Hospitalized With Bronchiolitis | Drug: 3% sodium chloride\|Drug: 0.9% sodium chloride | 227 | U.S. |
| NCT01768884 | yes | 01.01.16 | 27.07.17 | 18 | Evaluation of the Safety and Tolerability of Nitric Oxide (NO) Via Inhalation to Subjects With Bronchiolitis | Drug: Nitric Oxide\|Drug: Placebo | 43 | Israel |
| NCT01777347 | yes | 01.04.14 | 07.08.17 | 40 | Efficacy of 3% Hypertonic Saline in Acute Viral Bronchiolitis | Drug: 3% Saline\|Drug: 0.9% Normal Saline | 778 | France |
| NCT01812525 | yes | 01.04.16 | 05.09.19 | 41 | Moderate to Severe Bronchiolitis: Standard Therapy Versus Therapy With NaCl 3% Inhalations | Drug: NaCl 3%\|Other: Standard therapy | 120 | Switzerland |
| NCT01871857 | yes | 01.08.12 | 01.01.14 | 17 | 7% Hypertonic Saline for Acute Bronchiolitis | Drug: Hypertonic saline and epinephrine\|Drug: Normal saline and epinephrine | 101 | U.S. |
| NCT01873144 | yes | 01.12.12 | 01.06.14 | 18 | High Flow Therapy vs Hypertonic Saline in Bronchiolitis | Drug: Epinephrine 1/1000\|Drug: HSS 3%\|Device: HHHFNC\|Drug: NS (0.9%) | 75 | Spain |
| NCT02029040 | yes | 01.12.14 | 23.10.15 | 10 | Nebulized 3% Hypertonic Saline in the Treatment of Acute Bronchiolitis | Drug: 3% Hypertonic Saline\|Drug: 0.9 % normal saline | 52 | U.S. |
| NCT02094664 | yes | 01.06.15 | 18.03.19 | 45 | Heated Humidified Oxygen Compared to Dry Oxygen Therapy in Children With Bronchiolitis | Device: Heated and humidified oxygen | 32 | U.S. |
| NCT02126748 | yes | 01.01.15 | 02.06.16 | 17 | The Effectiveness of AAD and IPV to Treat Hospitalized Infants (<2years) With Acute Viral Bronchiolitis. | Other: inhalation 4ml hypertonic saline 3% 3x/day\|Device: Intrapulmonary Percussive Ventilation\|Procedure: Assisted Autogenic Drainage | 103 | Belgium |
| NCT02145520 | yes | 01.12.15 | 01.07.17 | 19 | Efficacy of Magnesium Sulfate in the Treatment of Bronchiolitis | Drug: Magnesium Sulfate\|Other: placebo | 200 | Qatar |
| NCT02162745 | yes | 01.05.14 | 01.03.16 | 22 | Nasal Irrigation in Infants With Bronchiolitis. | Drug: Isotonic solution (NaCl 0.9%)\|Drug: Hypertonic solution (NaCl 3%) | 133 | Italy |
| NCT02442427 | yes | 01.02.18 | 13.02.19 | 12 | Palivizumab Therapy for RSV-bronchiolitis | Drug: Palivizumab\|Other: Placebo | 420 | Qatar |
| NCT02458300 | yes | 01.03.15 | 01.11.19 | 56 | Clinical Evaluation of the Response to Chest Physiotherapy in Children With Acute Bronchiolitis | Other: Nebulization of hypertonic saline\|Other: Prolonged slow expiration technique (PSE)\|Other: Patient coughing Provocation (TP)\|Other: inspiratory maneuver to rhinopharyngeal cleaning DRR\|Other: Aspiration of secretions | 77 | Spain |
| NCT02460614 | yes | 01.12.13 | 01.12.16 | 36 | Effects of Rhinopharyngeal Retrograde Clearance in Children With Acute Viral Bronchiolitis | Procedure: Rhinopharyngeal clearance\|Procedure: Aspiration\|Other: 0.9% saline | 100 | Brazil |
| NCT02469597 | yes | 01.04.16 | 01.01.18 | 21 | Single Dose of Furosemide to Improve Respiratory Distress in Moderate to Severe Bronchiolitis | Drug: Furosemide\|Drug: Placebo | 46 | U.S. |
| NCT02602678 | yes | 01.01.16 | 01.02.19 | 37 | Prone Position Effects on Work of Breathing and Intrinsic PEEP in Children With Severe Acute Viral Bronchiolitis | Procedure: Supine then Prone position\|Procedure: Prone then supine position | 16 | France |
| NCT02618213 | yes | 01.05.18 | 20.04.17 | N/A | Comparison of High Flow Oxygenation Therapy and CPAP in Children With Bronchiolitis. | Device: Optiflow Junior\|Device: Continous Positive Airway Pressure | 50 | Denmark |
| NCT03171142 | yes | 01.05.17 | 01.01.18 | 8 | Effect of Heliox on RSV Bronchiolitis | Drug: Heliox\|Drug: Air | 104 | Egypt |
| NCT03342781 | yes | 01.03.17 | 18.06.18 | 15 | Using HFNC in Bronchiolitis | Device: high flow nasal oxygen therapy\|Device: Diffuser oxygen mask | 60 | Turkey |
| NCT03663660 | yes | 19.06.18 | 01.08.19 | 13 | Organizational Infrastructure Pediatric Plan in Bronchiolitis Epidemics | Other: Organizational infrastructure pediatric plan (OIPP) | 1636 | France |
| NCT03859947 | yes | 31.10.18 | 18.03.19 | 4 | High-flow Nasal Cannula Flow Rates, Severe Bronchiolitis | Device: Heated humidi•Âed high-flow nasal cannula | 168 | Turkey |
| NCT00355043 | no | 01.09.07 |  |  | Efficacy of Zinc in the Treatment of Bronchiolitis and Prevention of Wheezing Respiratory Illness in Children Less Than Two Years Old | Drug: Zinc sulphate 20 mg | 330 | Bangladesh |
| NCT00373802 | no | 01.06.05 |  |  | The Use of Nasal Phenylephrine in Infants With Bronchiolitis | Drug: Phenylephrine | 50 | U.S. |
| NCT00435994 | no | 01.12.12 |  |  | Assessment of Airway Obstruction in Infants With Lower Respiratory Infections | Drug: Inhaled primatene will be given as a breathing treatment\|Other: Nasal Washing | 59 | U.S. |
| NCT00863317 | no | 01.07.11 |  |  | Trial of Montelukast for Treatment of Acute Bronchiolitis | Drug: montelukast sodium\|Other: sucrose | 141 | U.S. |
| NCT01238848 | no | 01.05.12 |  |  | Efficacy of Nebulized Hypertonic Saline in the Treatment of Acute Bronchiolitis | Drug: Hypertonic saline\|Drug: Normal saline | 82 | Argentina |
| NCT01370187 | no | 01.06.11 |  |  | Montelukast for Acute Bronchiolitis and Postbronchiolitis Viral Induced Wheezing | Drug: Montelukast | 146 | Iran |
| NCT01460524 | no | 01.05.13 |  |  | Nebulized Hypertonic Saline for Bronchiolitis |  | 2580 | France |
| NCT01498094 | no | 01.12.14 |  |  | Study of High-flow Oxygen Therapy Against Standard Therapy in Bronchiolitis | Procedure: High Flow Nasal Cannula Oxygen Therapy\|Other: Standard low flow oxygen | 79 | Canada |
| NCT01834820 | no | 01.06.15 |  |  | Epinephrine, Dexamethasone, and Hypertonic Saline in Bronchiolitis, Randomised Clinical Trial of Efficacy and Safety | Drug: Epinephrine and Dexamethasone\|Drug: Hypertonic Saline\|Drug: Normal Saline | 120 | Mexico |
| NCT02233985 | no | 01.03.17 |  |  | Nebulized 3% Hypertonic Saline Solution Treatment of Bronchiolitis in Infants | Drug: 0.9% Sodium Chloride\|Drug: 3% Sodium Chloride | 64 | Mexico |
| NCT02538458 | no | 01.05.16 |  |  | Efficacy and Safety of 3% Hypertonic Saline Inhalation (24h vs 72h) to Treat Acute Bronchiolitis in Hospitalized Infants | Drug: 3 % hypertonic saline up to 72H\|Drug: 3 % hypertonic saline up to 24H | 120 | France |
| NCT02571517 | no | 01.12.13 |  |  | Glucocorticoid Therapy Impact on the Inflammatory Response and Clinical Evolution in Patients With Severe Bronchiolitis | Drug: Glucocorticoids\|Other: Placebo | 94 | Spain |
| NCT02791711 | no | 01.12.14 |  |  | Using Nasal Broadband Glasses in the Initial Management of Severe Bronchiolitis in Infants Admitted in ICU | Other: High Flow Nasal Cannula | 33 | France |
| NCT02853838 | no | 01.10.16 |  |  | Chest Physiotherapy in Infants Between 0 and 12 Months Old With Acute Bronchiolitis SRV(+) | Other: Prolonged slow expiration+provoked coughing\|Other: Manual chest wall vibration\|Other: Standard Therapy | 204 | Chile |
| NCT02856165 | no | 23.11.17 |  |  | High-flow Nasal Oxygen Therapy in Hospitalized Infant With Moderate-to-severe Bronchiolitis | Device: High-flow nasal canula oxygen therapy\|Device: Low-flow oxygen therapy with standard nasal canula | 268 | France |
| NCT03053388 | no | 28.04.18 |  |  | Evaluation of the Efficacy Safety and Tolerability of Nitric Oxide Given Intermittently Via Inhalation to Subjects With Bronchiolitis | Drug: Nitric Oxide\|Other: Supportive treatment | 69 | Israel |
| NCT03143231 | no | 31.05.17 |  |  | Normal Saline vs Hypertonic Saline in the Treatment of Bronchiolitis | Other: Normal saline\|Other: Hypertonic saline | 128 | Lebanon |
| NCT03614273 | no | 29.02.16 |  |  | Nebulized Hypertonic Saline (3%) Versus Nebulized Adrenaline for Treatment of Bronchiolitis | Drug: Hypertonic saline\|Drug: Adrenaline | 60 | India |
| NCT03799406 | no | 01.12.18 |  |  | Vitamin D Supplementation for Acute Bronchiolitis | Drug: Cholecalciferol\|Other: Placebo | 60 | Egypt |
