# Supplementary material for: Decision-making in acute viral bronchiolitis: A universal guideline and a publication gap
Source: PLoS One. 2020 Aug 18;15(8):e0237801. doi: 10.1371/journal.pone.0237801 (PMC7433885; doi:10.1371/journal.pone.0237801)
Supplement: Attachment — Submitted filename: Response to Reviewers_2020_07_29.docx [file pone.0237801.s002.docx]

**Response to Reviewers**

**Decision-making in acute viral bronchiolitis: a universal guideline and a publication gap**

We thank the reviewers for their thoughtful comments that were very helpful to further strengthen the manuscript. We are addressing the comments – numbered in consecutive order - in the following section point by point:

**Journal Requirements:**

Comment 1: Please ensure that your manuscript meets PLOS ONE's style

requirements, including those for file naming.

Answer 1: done.

Comment 2: Please include a caption for figure 4.

Answer 2: The caption was included in the results section (lines 148-149).

Comment 3: Please include captions for your Supporting Information files at the

end of your manuscript, and update any in-text citations to match

accordingly. Please see our Supporting Information guidelines for more

information: http://journals.plos.org/plosone/s/supporting-information

Answer 3: The caption was included at the end of the manuscript. In-text citations were checked and are appropriate. The file was named accordingly.

**Reviewer #1:**

Comment 4: Please check in results: patients numbers: Subjects mean age, comorbilities, because this is an important information for the treatment issue.

Answer 4: We thank the reviewer for this important comment. The patient numbers for published and unpublished studies, respectively, are given in the results section, lines 134-137. For three of the unpublished studies, preliminary results are posted on the ClinicalTrials.gov website. However, these data are very scarce. Mean age of the participants (4.5 ± 3.8 months; 8.2 ± 5.5 months; 11 ± 6 months) was not different from the mean age of the published studies and it was consistent with the typical population of pediatric patients with acute viral bronchiolitis. No information about comorbidities are available for any of the unpublished studies. We agree with the reviewers that patient numbers, patient age, and comorbidities may be substantial confounders important for clinical practice. However, from the available published data we are not able to provide further insight into this issue.

Comment 5: Line 135/136 You can improve the idea

Answer 5: We agree that this phrase was difficult to understand and added a word for clarification.

Comment 6: line 143, please check it. It is better to write 20 months

Answer 6: We agree with the reviewer and edited the respective phrase accordingly (line 143).

**Reviewer #2:**

Comment 7: This is a well-written manuscript demonstrating the publication gap regarding treatment of viral bronchiolitis following publication of the relevant 2014 AAP Clinical Practice Guideline. Based on recommendations included in the Clinical Practice Guideline, most traditional treatment interventions are discouraged and discharge to home is encouraged if the infant does not show any signs of bacterial infection, has adequate po intake and there is only mild or no hypoxemia. Several of the recommendations are supported by low-quality evidence.

One characteristic example that the authors need to mention in their Discussion is the recommendation not to administer supplemental oxygen if SpO2 >90% (Expert Opinion; Evidence quality D). Many infants with bronchiolitis upon discharge from the hospital have lower basal SpO2 than healthy control infants and an elevated oxygen desaturation index during sleep (Kaditis et al. Infants with viral bronchiolitis demonstrate two distinct patterns of nocturnal oxyhaemoglobin desaturation. Acta Paediatrica 2015; 104: e106-111). The pathophysiological and clinical consequences of mild nocturnal hypoxemia and especially of the intermittent type are unknown.

The consequence of the publication gap in the field is that many clinicians use interventions that are not recommended by the AAP Guidleine like inhaled bronchodilators and supplemental oxygen.

Answer 7: We thank the reviewer for this comment. We agree and added a paragraph and a reference into the discussion section.
